# Intragenic duplication in the *PHKD1* gene in autosomal recessive polycystic kidney disease

**DOI:** 10.1186/s12881-015-0245-3

**Published:** 2015-10-26

**Authors:** Jun Miyazaki, Mayuko Ito, Haruki Nishizawa, Takema Kato, Yukito Minami, Hidehito Inagaki, Tamae Ohye, Masafumi Miyata, Hiroko Boda, Yuka Kiriyama, Makoto Kuroda, Takao Sekiya, Hiroki Kurahashi, Takuma Fujii

**Affiliations:** Division of Molecular Genetics, Institute for Comprehensive Medical Science, Fujita Health University, 1-98 Dengakugakubo, Kutsukake-cho, Toyoake, Aichi 470-1192 Japan; Department of Obstetrics and Gynecology, Fujita Health University School of Medicine, 1-98 Dengakugakubo, Kutsukake-cho, Toyoake, Aichi 470-1192 Japan; Genome and Transcriptome Analysis Center, Fujita Health University, Aichi, Japan; Department of Genetic Counseling, Fujita Health University Hospital, Aichi, Japan; Department of Pediatrics, Fujita Health University School of Medicine, 1-98 Dengakugakubo, Kutsukake-cho, Toyoake, Aichi 470-1192 Japan; Department of Diagnostic Pathology, Fujita Health University School of Medicine, 1-98 Dengakugakubo, Kutsukake-cho, Toyoake, Aichi 470-1192 Japan

**Keywords:** ARPKD, Prenatal diagnosis, Target exome, *PKHD1*, Duplication

## Abstract

**Background:**

In the present study, we report on a couple who underwent prenatal genetic diagnosis for autosomal recessive polycystic kidney disease (ARPKD).

**Case presentation:**

This healthy couple had previously had a healthy boy but had experienced two consecutive neonatal deaths due to respiratory distress resulting from pulmonary hypoplasia caused by oligohydramnios. The woman consulted our facility after she realized she was pregnant again. We promptly performed a carrier test for the *PKHD1* gene by target exome sequencing of samples from the couple. A pathogenic mutation was identified only in the paternal allele (c.9008C>T, p.S3003F). The mutation was confirmed by Sanger sequencing of the DNA from formalin-fixed, paraffin-embedded, kidney tissue of the second neonate patient and was not found in the healthy sibling. We then performed haplotype analyses using microsatellite markers scattered throughout the *PKHD1* gene. DNA from the amniocentesis was determined to belong to a carrier, and the couple decided to continue with the pregnancy, obtaining a healthy newborn. Subsequent detailed examination of the exome data suggested higher read depth at exons 45 and 46. Multiplex ligation-dependent probe amplification allowed identification of duplication of these two exons. This case suggests the potential usefulness of target exome sequencing in the prenatal diagnosis of the *PKHD1* gene in ARPKD.

**Conclusions:**

This is the first report of intragenic duplication in the *PKHD1* gene in ARPKD.

**Electronic supplementary material:**

The online version of this article (doi:10.1186/s12881-015-0245-3) contains supplementary material, which is available to authorized users.

## Background

Autosomal recessive polycystic kidney disease (ARPKD) is recognized as a severe hereditary form of polycystic kidney disease [MIM 263200]. Patients present with enlarged kidneys with dilatations of the collecting ducts and congenital hepatic fibrosis. Severely affected neonates have oligohydramnios and pulmonary hypoplasia that cause respiratory distress in the perinatal period. Approximately 30 % of affected children die within the first year of life [1]. Survivors of the perinatal respiratory insufficiency and cases with later onset generally progress to end-stage renal disease before adulthood. A minority of patients come to medical attention in adulthood with liver-related complications and mild kidney disease. ARPKD is a rare disorder that affects ~1 in 20,000 live births.

ARPKD is caused by mutations in the *PKHD1* gene, chromosomally located at 6p12.2 [2, 3]. The *PKHD1* gene is mainly expressed in the kidneys and liver, as well as the pancreas. The encoded fibrocystin/polyductin protein is a receptor-like transmembrane protein that localizes at primary cilium, particularly at the basal body of the cilium, and possibly functions as the sensory antenna in renal epithelial cells or biliary duct cells [4]. Biallelic truncating mutations or pathogenic missense mutations are identified in most ARPKD cases [5]. The *PKHD1* gene is considered the only gene with mutations causally associated with ARPKD.

The carrier test for ARPKD has conventionally been performed by Sanger sequencing of the *PKHD1* gene. However, this process is laborious and time-consuming because the *PKHD1* gene is a large gene spanning a 470-kb genomic region and consisting of 86 exons, including alternatively spliced exons [2, 3]. In prenatal diagnosis, each mutation of the carrier parents needs to be determined within a short period. Denaturing high-performance liquid chromatography is one sequencing option to reduce time and cost, although it is difficult to find the conditions for optimal sensitivity [6, 7]. Recent advances in next-generation sequencing have allowed *PKHD1* mutations to be screened with exome sequencing [8]. In our present report, we describe carrier testing using target exome sequencing performed for prenatal diagnosis. In addition, we report a rare intragenic duplication in the *PKHD1* gene in ARPKD.

## Methods

### Human samples

**Fig. 1:**
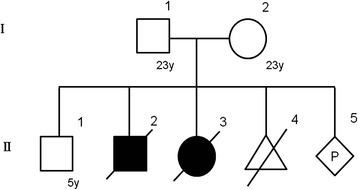
Family pedigree of the ARPKD family

In this study, we used genomic DNA samples from members of a Japanese family with ARPKD siblings (Fig. 1). After informed consent was provided, peripheral blood samples were obtained. Genomic DNA was purified by QuickGene-610 L (Fuji Film). In addition, we extracted DNA from formalin-fixed, paraffin-embedded (FFPE), kidney tissue from one of the ARPKD neonatal patients with the aid of QIAamp DNA FFPE Tissue (Qiagen). This study was approved by the Ethical Review Board for Human Genome Studies of Fujita Health University (accession number 13-14; approved on September 24, 2013).

### Next-generation sequencing

For target exome sequencing, libraries were prepared using the TruSight Rapid Capture kit (Illumina) according to the manufacturer’s specifications. After tagmentation with transposase, the libraries were amplified to add indices and common adapters for subsequent cluster generation and sequencing. The libraries were quantified by a 2100 Bioanalyzer (Agilent Technologies) using the High Sensitivity DNA Kit (Agilent Technologies, 5067–4626). Next, exon capture was performed using a TruSight Inherited Disease Panel (Illumina, TG141–1005). Prior to cluster generation, the libraries were further quantified by Qubit (Invitrogen, Q32866) using the Qubit dsDNA HS Assay Kit (Invitrogen, Q32851). Finally, the prepared library was loaded on an Illumina MiSeq clamshell-style cartridge for paired-end sequencing (Illumina). The data were analyzed with the aid of Variant Studio for filtering and annotation (Illumina). The reads were reanalyzed for copy number alterations using the Comparative Exome Quantification analyzer CEQer [9].

### Microsatellite analyses

A total of seven microsatellite markers were selected based on previous reports (Table 1) [10, 11]. Forward primers were labeled with FAM. The PCR products were analyzed by capillary electrophoresis (ABI3730 Genetic Analyzer; Applied Biosystems).

**Table 1:** PCR primers used for microsatellite analysis

Marker	Forward primer (FAM)	Backward primer
D6S465	GTCCAGAAGGGAATTTTCTACTCTTTG	CTTTTCAATCATATAACTTTAAAAATGCC
3–204.2 k	GCGTTGACCTATTTCTACACAG	CTTAGGCAAATAAGACCTGGAGAGG
D6S1714	TGTATCCACTGCCATCACTT	AGCACCAAATGACACAGAAC
D6S243	AATAGAACAAATTTGGCCTCTGG	CATCCTTAGAATGAAAAATTACTCAGG
MBC-2 (D6S0919i)	CATGAGGTGAGAGTGAGAAGAGC	AAAGCCAGTTTCCTGACAC
D6S1344	AGCCCTGTGGTTATTTATGCTTCTC	GGTTGTTCCTTCTCTGAACATGGCCC
5–326 k (D6S0460i)	CCTACCCTCTAAAAGGATCTGGG	CCCCACCTACCAACTCTGAATAAA

### Multiplex ligation-dependent probe amplification

Multiplex ligation-dependent probe amplification (MLPA) probe pairs for the *PHKD1* gene were used (SALSA MLPA probe mix P341/P342; MRC-Holland, Amsterdam, the Netherlands). In this approach, the MLPA probes consist of two oligonucleotides, each containing a PCR primer sequence and a variable length sequence complementary to the target. Genomic DNA was denatured (1 min at 98 °C) and subsequently hybridized to the MLPA probe pairs in accordance with the manufacturer’s protocol. After ligation, probe pairs were amplified using universal primers. The multiplex PCR products were then separated on a capillary sequencer (ABI3730 Genetic Analyzer).

### PCR amplification of the junction fragments

To isolate junction fragments, PCR was performed using ExTaq (TaKaRa, Shiga, Japan). The PCR conditions were 35 cycles of 10 s at 98 °C and 3.5 min at 60 °C. PCR primers were designed using sequence data from the human genome database. The following primers were used for amplification: PKHD1_Ex45F, 5′-CAAACTGTGA AGCTCTGGAA CAGAG-3′, and PKHD1_Ex46R, 5′-GCAAATACTT CAGTTACTGA CAGC-3′. The resulting PCR products were checked on 1 % agarose gels. To obtain smaller PCR products, PCR was performed with the following primers: PKHD1_Intron44, 5′-GCACAGGAAC ATCACCCAAT CTCCAAC-3′, and PKHD1_Intron46-2, 5′-CGGTGCTGTT TACCGTACCC TC-3′. The PCR conditions were 35 cycles of 10 s at 98 °C, 30 s at 60 °C, and 30 s at 72 °C. The PCR products were subjected to ExoSAP-IT digestion (Affymetrix) and then sequenced bidirectionally by capillary electrophoresis (ABI3730 Genetic Analyzer). Breakpoint sequences were characterized using RepeatMasker (http://www.repeatmasker.org/) and non-B DB (https://nonb-abcc.ncifcrf.gov/apps/site/default).

## Case presentation

A healthy couple, both 23 years old, consulted our facility after they noticed their fifth pregnancy at 4 weeks of gestation. Their first pregnancy was uncomplicated, and they had a healthy boy (Fig. 1). The second pregnancy was complicated by oligohydramnios. At 1 week before the due date, the woman vaginally delivered a boy, who died at the second day after birth from respiratory distress due to pulmonary hypoplasia. The woman subsequently got pregnant and the pregnancy was again complicated by oligohydramnios. At 1 month before the due date, the woman delivered a girl via Caesarian section due to breech presentation, who also died at the second day after birth due to respiratory distress. An autopsy was performed and multiple cysts were observed in both the liver and kidneys. The neonate was thus diagnosed with ARPKD. The fourth pregnancy ended in an early pregnancy loss. Soon after they noticed the fifth pregnancy, they consulted our facility to undergo prenatal genetic testing for ARPKD. The couple was screened by abdominal ultrasound and no polycystic kidney disease was observed.

## Consent

Written informed consent was obtained from the patient for publication of this manuscript and any accompanying images. A copy of the written consent is available for review by the Editor of this journal.

## Results

### Carrier testing by target exome sequencing

**Fig. 2:**
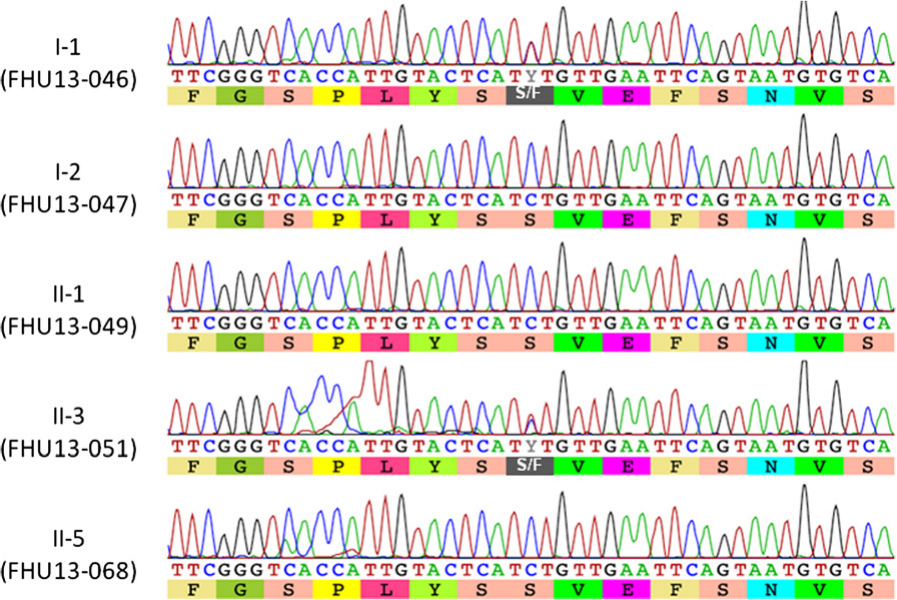
Pathogenic nucleotide alteration of the *PKHD1* gene identified in the paternal allele. Sample names are indicated on the left: FHU14–046 (I-1, father), FHU13–047 (I-2, mother), FHU13–049 (II-1, healthy sibling), FHU13–051 (II-3, proband), and FHU13–068 (II-5, amniotic fluid)

We performed carrier testing using target exome sequencing of genomic DNA only from both parents because the DNA of the proband obtained from the FFPE sample was too degraded to produce unbiased sequence data. A missense mutation, c.9008C>T (p.S3003F), was identified in exon 58 of the *PKHD1* gene in the paternal genome. Sanger sequencing was performed and the presence of the mutation was confirmed (Fig. 2). The DNA from the FFPE tissue also carried the p.S3003F mutation, whereas the mother and healthy sibling did not. This mutation has not been reported in the human genomic database. The position of the mutated amino acid is at the highly conserved region of the extracellular domain just before the transmembrane domain (phastCons = 0.901, cons score GERP = 5.67). The SIFT score is 0 (deleterious) and PolyPhen 2 score is 0.984 (probably damaging), suggesting a pathogenic mutation. On the other hand, no possible pathogenic mutation was identified in the maternal genome.

### Prenatal diagnosis by haplotype analyses

**Fig. 3:**
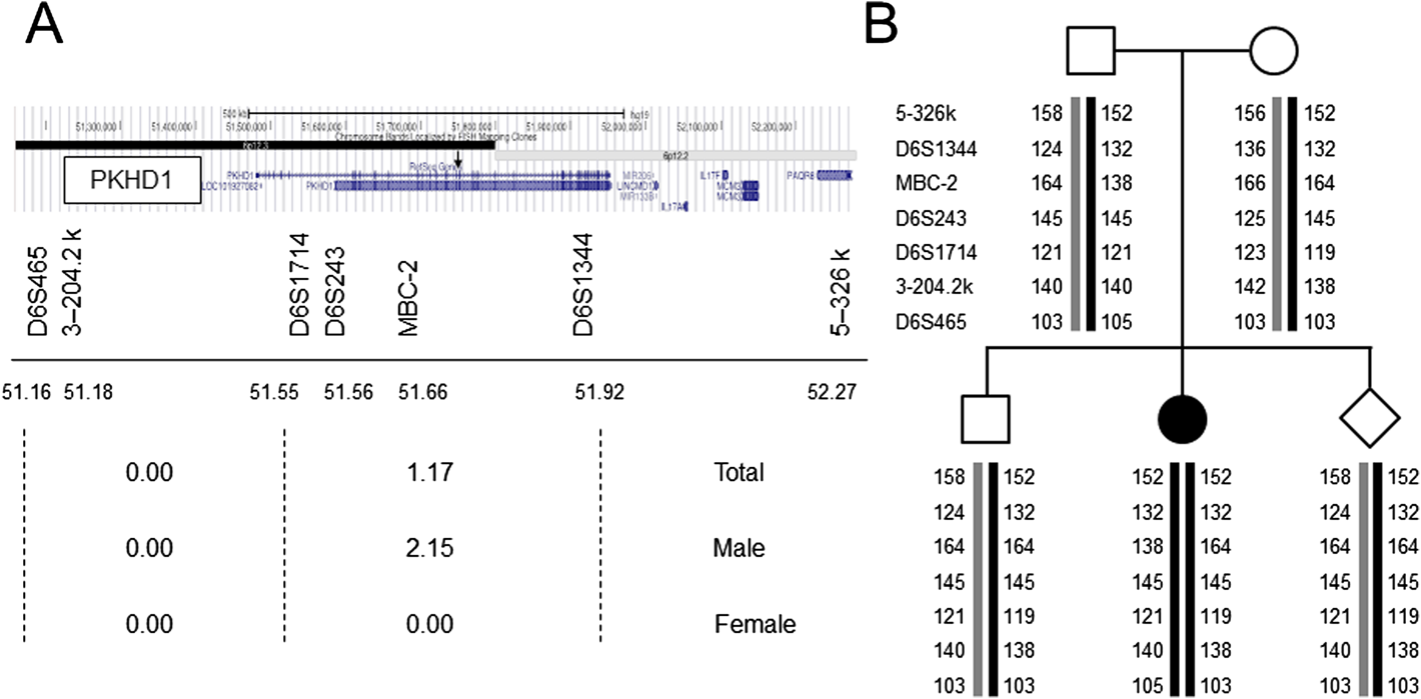
Microsatellite analysis of the disease haplotype. **a** Genomic structure of the region around the *PKHD1* gene. Seven microsatellite markers were used. The upper panel indicates a transcription map of this region with the name of the seven markers at the bottom. Physical distances (middle panel) as well as genetic distances (lower panel) between the markers are indicated. The location of exons 45 and 46 (arrow) are between MBC-2 and D6S1344. **b** Haplotype analysis of the family members. The numbers indicate the size of the PCR products

Since a maternal mutation was not identified, we decided to perform prenatal diagnosis by haplotype analysis. We selected seven microsatellite markers encompassing the entire *PKHD1* gene, including upstream and downstream regions (Fig. 3a). By analyses of DNA from the parents, proband, and the healthy sibling, the disease haplotype was successfully determined (Fig. 3b). The healthy sibling was found to be a carrier of the maternal disease haplotype. We then performed an amniocentesis at 17 weeks of gestation. The DNA isolated from amniotic fluid was analyzed for the genotype of these microsatellite markers, and the results of the typing showed that the fetus was a carrier of the maternal disease haplotype but not of the paternal pathogenic haplotype (Fig. 3b). The couple decided to continue the pregnancy and finally obtained a healthy newborn.

### Intragenic duplication identified by MLPA

**Fig. 4:**
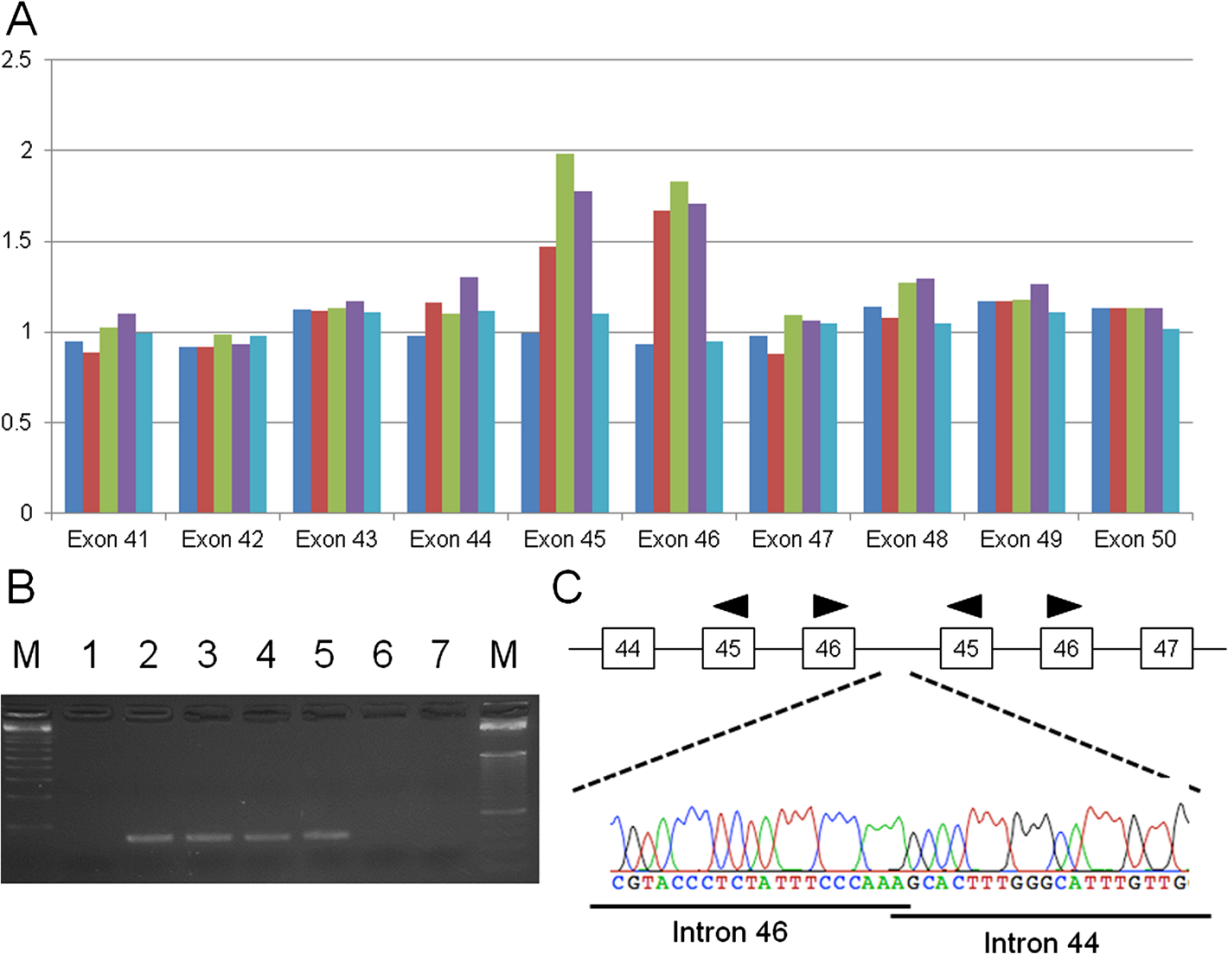
Exon duplication of the *PKHD1* gene identified in the maternal allele. **a** Diagram of the MLPA results. Dark blue box, FHU14–046 (I-1, father); red box, FHU13–047 (I-2, mother); green box, FHU13–049 (II-1, healthy sibling); purple box, FHU13–068 (II-5, amniotic fluid); light blue box, normal healthy control. **b** Results of junction PCR analysis. Lane M, size markers; lane 1, FHU14–046 (I-1, father); lane 2, FHU14–047 (I-2, mother); lane 3, FHU13–049 (II-1, healthy sibling); lane 4, FHU13–051 (II-3, proband); lane 5, FHU13–068 (II-5, amniotic fluid); lane 6, normal healthy control; lane 7, water control. **c** Results of Sanger sequencing of the junction PCR product. The upper panel indicates the exon-intron structure around the junction

We subsequently reanalyzed the target exome sequencing data, focusing on read depth to identify deletion or duplication mutations in the maternal genome. The CEQer algorithm revealed that the read depths of exons 45 and 46 were higher than those of other exons (Additional file 1: Figure S1). We then performed MLPA for all exons of the *PKHD1* gene using genomic DNA from both parents as well as a normal control. The amounts of PCR products for exons 45 and 46 in maternal DNA were 1.5 times higher than those from the father or normal control (Fig. 4a). A similar finding was also observed in the DNA from the healthy sibling. This duplication is not found in the database of copy number variations. We considered that the maternal mutation was a duplication of exons 45 and 46. If the duplication is a tandem repeat, it results in a frameshift that produces a pathogenic C-terminally truncated protein.

To analyze the breakpoint of this duplication at a nucleotide resolution, we designed inversely oriented PCR primers within both exons 45 and 46. Long-range PCR using primers successfully yielded a PCR product that incorporated the junction of the tandem duplication. Multiple PCR primers were then designed upstream and downstream of the putative breakpoint (introns 44 and 46) and PCR-direct sequencing was performed (Fig. 4b). Sequence analysis of the PCR product revealed the following: rsa[hg19] 6p12.3(51,746,249–51,751,855) × 3. The proximal and distal region was joined with a one-nucleotide microhomology (Fig. 4c).

We further analyzed the sequence around the proximal and distal breakpoint regions. Both of the breakpoints in introns 44 and 46 were located near the LINE1 element, whereas no substantial homology was found between the breakpoint regions (data not shown). We did not identify any non-B DNA motif that could have induced replication fork stalling at either the proximal or distal breakpoint regions [12].

## Discussion

In our present study, we used target exome sequencing as a carrier test for the prenatal diagnosis of ARPKD. In ARPKD, genetic screening of the *PKHD1* gene is typically performed by PCR amplification of all coding exons followed by Sanger sequencing. This method is laborious and time-consuming however because the *PKHD1* gene is a large gene spanning a 470-kb genomic region and consisting of 86 exons, including alternatively spliced exons, and recently new exons were identified further [2, 3, 13]. For prenatal diagnosis, each mutation of the carrier parents needs to be determined within a short period. Target exome sequencing can overcome these issues. Because the sequence of all coding exons can be obtained within a week, this strategy would be particularly useful when a carrier couple consults a medical facility for a prenatal diagnosis after they notice their pregnancy.

In ARPKD, genetic screening of the *PKHD1* gene by PCR-direct sequencing of all coding exons can identify disease-causing mutations in up to 82 % of cases [14]. Since most mutations identified by this method are nucleotide alterations, the remaining 18 % might carry copy number mutations that can escape this screening. One report has stated that MLPA screening did not identify any exon deletion/duplication mutation in 39 ARPKD families [5]. Two previous case reports have described multiple exon deletions in the *PKHD1* gene [15, 16]. To our knowledge, our current study is the first report of a duplication mutation in the *PKHD1* gene in a patient with ARPKD. Quantitative analysis of the exome sequence data provided a clue to the duplication which was successfully identified by subsequent MLPA. Since target exome analysis may identify a mutation at a nucleotide resolution as well as the copy number variation of the exons, target exome sequencing could replace PCR-direct sequencing or other related PCR-based screening techniques in the near future.

The junction of the duplication showed one-nucleotide identity between the breakpoints without extensive homology, suggesting that fork stalling and template switching or microhomology-mediated breakage-induced replication is a plausible mechanism its formation [17]. Since we found no sequence motif susceptible to the formation of a non-B structure, replication stalling might have occurred in a sequence-independent fashion. It has also been speculated that two introns are located at close proximity in the nucleus at the timing of the replication stall in either of the introns [18].

We identified the duplication junction at a nucleotide resolution, which allowed us to detect the pathogenic allele by simple PCR. In our current case, the duplication mutation was identified after the delivery of the healthy newborn. However, if careful examination of exome data had enabled the identification of both the paternal mutation and maternal duplication, we could have performed prenatal diagnosis directly by detection of both paternal and maternal mutations. In the case of an amniocentesis, the sample often contains a considerable amount of dead cells that impede copy number analysis techniques such as cytogenetic microarray and MLPA. The determination of the breakpoint would provide a simple PCR-based diagnosis of the pathogenic allele. We thus conclude that target exome sequencing of the *PKHD1* gene has utility as a carrier test for the prenatal diagnosis of ARPKD.

## Conclusion

This case suggests the potential usefulness of target exome sequencing in the prenatal diagnosis of the *PKHD1* gene in ARPKD. This is the first report of intragenic duplication in the *PKHD1* gene in ARPKD.

## Additional file

Additional file 1: Figure S1. Quantitative analysis of the exome data using the Comparative Exome Quantification analyzer (CEQer). The upper panel indicates the map of the *PKHD1* gene. The lower panel indicates the normalized read depth. (TIFF 93 kb)

## Abbreviations

ARPKD: Autosomal recessive polycystic kidney disease
FFPE: Formalin-fixed, paraffin-embedded
MLPA: Multiplex ligation-dependent probe amplification
