# Supplementary figures and images for: Intragenic duplication in the PHKD1 gene in autosomal recessive polycystic kidney disease
Source: BMC Med Genet. 2015 Oct 26;16:98. doi: 10.1186/s12881-015-0245-3 (PMC4623244; doi:10.1186/s12881-015-0245-3)

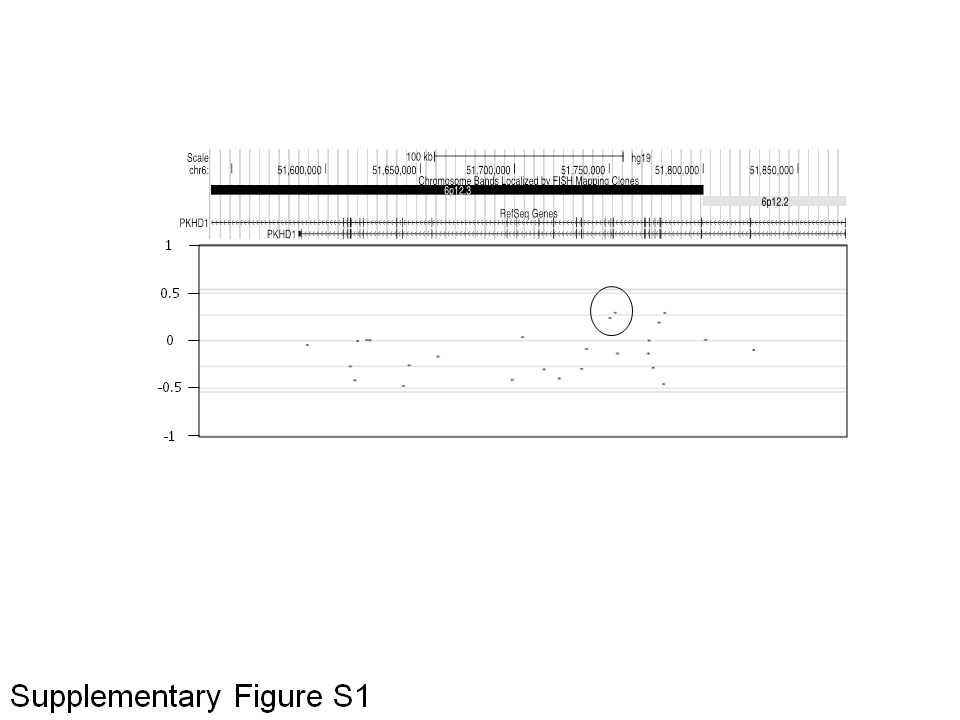

Supplement: Additional file 1: Figure S1. — Quantitative analysis of the exome data using the Comparative Exome Quantification analyzer (CEQer). The upper panel indicates the map of the PKHD1 gene. The lower panel indicates the normalized read depth. (TIFF 93 kb) [file 12881_2015_245_MOESM1_ESM.tiff]
